# Intraspecific variation for host immune activation by the spider mite *Tetranychus evansi*

**DOI:** 10.1098/rsos.230525

**Published:** 2023-06-14

**Authors:** Jéssica Teodoro-Paulo, Juan M. Alba, Steven Charlesworth, Merijn R. Kant, Sara Magalhães, Alison B. Duncan

**Affiliations:** ^1^ cE3c—Centre for Ecology, Evolution and Environmental Changes & CHANGE—Global Change and Sustainability Institute, Faculty of Sciences, University of Lisbon, Lisbon, Portugal; ^2^ Institute for Biodiversity and Ecosystem Dynamics, University of Amsterdam, Amsterdam, The Netherlands; ^3^ Institut des Sciences de l’Évolution, University of Montpellier, CNRS, IRD, Montpellier, France

**Keywords:** inbred lines, defence suppression, plant–herbivore interactions, host–parasite interactions

## Abstract

Many parasites can interfere with their host's defences to maximize their fitness. Here, we investigated if there is heritable variation in the spider mite *Tetranychus evansi* for traits associated with how they interact with their host plant. We also determined if this variation correlates with mite fecundity. *Tetranychus evansi* can interfere with jasmonate (JA) defences which are the main determinant of anti-herbivore immunity in plants. We investigated (i) variation in fecundity in the presence and absence of JA defences, making use of a wild-type tomato cultivar and a JA-deficient mutant (*defenseless-1*), and (ii) variation in the induction of JA defences, in four *T. evansi* field populations and 59 inbred lines created from an outbred population originating from controlled crosses of the four field populations. We observed a strong positive genetic correlation between fecundity in the presence (on wild-type) and the absence of JA defences (on *defenseless-1*). However, fecundity did not correlate with the magnitude of induced JA defences in wild-type plants. Our results suggest that the performance of the specialist *T. evansi* is not related to their ability to manipulate plant defences, either because all lines can adequately reduce levels of defences, or because they are resistant to them.

## 1. Introduction

Antagonistic interactions between organisms, such as between parasites and their hosts, are abundant in nature. Hosts have evolved numerous traits relating to resistance or avoidance to minimize the negative effects on fitness caused by parasites [1–4]. This, in turn, selects for parasites that can overcome, resist or interfere with host resistance [5–7], which can lead to the maintenance of genetic variation in host and parasite populations [8].

Interference of host defences is a strategy that has evolved in diverse plant parasites, including viruses [9], nematodes [10], lepidopteran larvae [11], mites [12–14] and aphids [15]; and animal parasites, such as parasitoid wasps [16], *Plasmodium* [17] and HIV [18]. Immune interference is often associated with the production of molecules by the parasite that alter host–cell structure and function (in plants [19]; in animals [20]) referred to as ‘effectors'. Many effectors have been identified and some of these have been characterized in detail (reviewed in [21]), but for many the mode of action, genetic variation and their costs are unclear. Theory predicts that their production may incur direct physiological costs for the parasite [22]. There may also be ecological costs due to parasite-mediated changes in host immune traits, as a disarmed host may be more suitable for competing parasites (e.g. helminths [23]; reviewed for spider mites [24]; reviewed in [25]; theoretically shown in [26]). These physiological and ecological costs may be responsible for the maintenance of genetic diversity for immune interference of host defences [27], although evidence for this is still scarce [9,16,28].

Plant immunity largely depends on the upstream action of plant hormones, with salicylic acid (SA) and jasmonic acid (JA) being the central players in mediating defences such as the production of toxic secondary metabolites and proteins that interfere with herbivore digestion and development [29–31]. The SA pathway is mainly involved in the response against biotrophic pathogens and phloem-feeding herbivores, while the JA pathway is involved in the defence against necrotrophic pathogens and chewing or cell-content-feeding herbivores [31]. Several herbivores have been shown to interfere with these defence pathways and this has been linked to increases in arthropod fitness (e.g. weight, fecundity or survival; lepidopteran larvae [11]; white flies [32]; aphids [33,34]; spider mites [12,13,35]). In some cases, interference occurred after the initial onset of induced defences [13,36].

There is evidence that plant defence interference (e.g. spider mites [37]; aphids [38]) and effector production by herbivores (spider mites [39,40]; Lepidoptera [41]) are plastic traits that can be influenced by environmental cues such as competition [37], light–dark cycles [42] or temperature [41]. This plasticity may serve to limit physiological or ecological costs associated with immune interference. For instance, the arrival of competitors has been shown to modulate immune interference in spider mites, by stronger suppression of local defences in plant leaves [37]. In addition, mites were also shown to shield their feeding site from competitors via physical barriers (through webbing [43], or by using leaf hairs as a refuge [44]), and to mitigate competitive population growth via reproductive interference [45]. This may act to buffer competitor-driven selection against immune interference [24].

Several studies have reported immune interference (often referred to as defence suppression) in tetranychid spider mites [12,13,39,40,43]. Knegt *et al*. [46] observed levels of immune interference to differ among *T. evansi* populations collected on different continents. In this study, we measure intraspecific variation in immune interference at the population and genotype level, and how it relates to fitness differences. We used four field populations collected in Portugal and 59 inbred lines generated from an outbred population, created from controlled crosses of all four field populations [44], thus capturing the genetic variation present in them. First, we compared the fecundity of the four field populations and inbred lines on a common tomato variety (referred to as wild-type, WT) and on a mutant tomato unable to activate the JA pathway (*defenseless-1*, *def-1*) [47–49] to assess how they cope with JA defences. We then assessed the magnitude of induced defences in the WT for 19 lines using marker genes involved in the mite-induced JA and SA pathways. We observed that fecundity across the four field populations differed marginally, with no difference in the expression of the tomato defence genes. Yet we did find variation in fecundity and their effect on the expression of defence genes across the lines, with some lines inducing immune responses. This variation in fecundity has a genetic basis but did not correlate with their effect on induced tomato defences.

## 2. Methods

### 2.1. The study system

*Tetranychus evansi* is an arrhenotokous mite species, feeding mostly on Solanaceae plants [50]. In the laboratory, this species has a life cycle of approximately 13 days (egg to adult) at 25°C [51], with all life-stages residing on the host plant. We performed the experiments on tomato plants (*Solanum lycopersicum* L.) cv. Castlemart (WT) and the mutant *defenseless-1* in the Castlemart genetic background (*def-1*) [48]. *def-1* is unable to mount JA-related defences. All plants were grown in a climatic chamber (photoperiod of 16 : 8 h, 25:18°C, day:night, 50–60% relative humidity). Infestations with *T. evansi* occurred when plants were 28 days old, with four fully expanded leaves. All mite strains, prior to and for the experiments, were maintained in a temperature-controlled room (photoperiod of 16 : 8 h, 23.5 ± 2°C, 70% RH).

### 2.2. *Tetranychus evansi* field and outbred populations

The collection of the four field populations and the generation of the outbred mite population are described in detail in [44]. In summary, at four locations in Portugal in 2017 *T. evansi* mites were collected from field-grown tomato plants. We refer to these four mite populations as VIT, 6M1, QG and PBS. All four populations had the same ITS haplotype [44]. To remove *Wolbachia*, these populations were heat shocked at 33°C for 42 days and then used to create an outbred laboratory population, with a maximal level of genetic diversity, by performing controlled crosses while avoiding over-representation of genotypes from a given population [44]. Field and outbred populations were maintained on detached tomato leaves (28 days old, cv. Moneymaker) with the petioles submerged in water. All the females used in the experiment were obtained from cohorts of 100 mated females maintained on tomato cv. Moneymaker leaves.

### 2.3. *Tetranychus evansi* inbred lines

The generation of the 59 inbred mite lines, each originating from the outbred population, is described in detail in [44]. Brother–sister mating was imposed for each line for 15 generations resulting in 59 lines giving an expected level of 93.6% homozygosity [44]. All lines were maintained on detached tomato leaves (28 days old, cv. Moneymaker) placed on water-saturated cotton wool in 10 cm diameter Petri dishes. All the females used in the experiment were obtained from cohorts of 60 mated females maintained on tomato cv. Moneymaker leaves.

### 2.4. Benchmark mite strains

For all our experiments, *T. urticae* Santpoort-2 (‘KMB' in [35]) and *T. evansi* Baker & Pritchard Viçosa-1 [43] were used as benchmark controls for the induction and suppression of plant defences, respectively. These lines are referred to as the ‘inducer' and ‘suppressor' benchmarks as they have been shown in previous experiments to induce and suppress JA defences in tomato, respectively (e.g. [12,13,43]). The *T. urticae* strain was maintained on detached bean leaves (10 days old, cv. Speedy), and the *T. evansi* Viçosa-1 population on detached tomato leaves (28 days old, cv. Castlemart). Note that it was not possible to use a strain of *T. evansi* as a control for immune induction as this trait has not been found in this species.

### 2.5. The populations

#### 2.5.1. Fecundity of *Tetranychus evansi* field and outbred populations on WT and *def-1* tomato plants

First, we assessed fecundity in the four field populations and the outbred population in the presence (WT) or absence of functional JA-mediated defences (*def-1*). To this end, 25 mated females (15 ± 1 days old) from each of the *T. evansi* populations were placed on one non-terminal leaflet of one fully expanded leaf of WT or *def-1* tomato plants (day 0). Mite dispersal was prevented by isolating the adaxial surface of this leaflet with a 1 : 1 mix of entomological glue (Tanglefoot, The Scotts Company LLC, OH, USA), and lanolin (Sigma-Aldrich, St Louis, MO, USA), distributed around the adaxial edge of the leaflet. For each population and replicate, individual plants were used. The number of surviving females and their eggs were assessed four days after infestation. With these two measures, we calculated fecundity per female assuming linear mortality [52] by using [total eggs]/[(alive females + total females)/2] and using these numbers as the average per female. This equation accounts for differential female mortality, which is measured at the end of the assay and thus enables a more accurate representation of per capita fecundity. For each mite population, we included 8–11 replicates (i.e. one leaflet per plant). Assays for WT and *def-1* plants were performed separately, and done in three or four temporal experimental blocks, respectively.

#### 2.5.2. Expression of salivary effectors and tomato defences after infestation with the field and outbred populations

We measured the expression of salivary *effector 84* (*Te84* and *Tu84* in [14]) in the different spider mite populations and of genes implicated in JA defences (*Proteinase Inhibitor IIc* (*WIPI-IIc*) and *Proteinase Inhibitor IIf* (*WIPI-IIf*) [13]) in WT plants infested with mites. The mite *Ribosomal protein 49* (*RP49*) and the tomato *Actin* were used as housekeeping reference genes for spider mites and tomato plants, respectively (see electronic supplementary material, table S1, for primer sequences). For this, we used the leaflets from WT plants from the performance assay described above. In parallel, we also infested plants with the *T. urticae* inducer benchmark strain and the *T. evansi* immune suppressor benchmark strain in the same way as described above to have a quantitative reference for induction and suppression [13]. The part of the leaflet with glue and lanolin was discarded and the rest of the leaflet (plant and mite material) was collected, flash-frozen in liquid nitrogen and stored at −80°C for RNA extraction. For each mite population (field and outbred), we included 5–11 replicates. Uninfested plants (i.e. without mites) were used as a control.

Total RNA from the sampled WT leaves was isolated using a protocol adapted from Verwoerd *et al.* [53]. Our protocol differs from it in that: (i) we used phenol at room temperature and (ii) the 5 min sample incubation step was completed at room temperature. Next, 2 µg of RNA was DNAse-treated with Ambion Turbo DNA-free kit (Thermo Fisher Scientific, Waltham, MA, USA) and cDNA was synthesized with RevertAid H Minus Reverse Transcriptase (Thermo Fisher Scientific). Next, 1 µl of 10× diluted cDNA was used as a template for a 20 µl quantitative reserve-transcriptase polymerase chain reaction (RT-qPCR) using the SsoFast EvaGreenSupermix (Bio-Rad, Hercules, CA, USA) and the CFX96 Real-Time system (Bio-Rad). Gene expression was normalized using the ΔCt method [13] and, for graphical representation, scaled to the value with the lowest normalized average expression per gene.

#### 2.5.3. Impact of infestation with the field and outbred populations on *Tetranychus urticae* Santpoort-2 and host immune responses

Immune interference can be investigated by comparing the fecundity of a mite strain negatively affected by JA defences (*T. urticae* inducer Santpoort-2) on plants previously exposed to spider mites that suppress defences, with their fecundity on clean plants (i.e. not having had their defences suppressed) [35]. We used this method to assess the immune interference of the four field populations and the outbred population.

We first infested WT plants with females from the field or outbred population for four days and then removed adults and eggs. Next, cleaned leaflets were re-infested with three *T. urticae* inducer mated females (15 ± 1 days old). Female survival and the number of eggs of the *T. urticae* inducer strain were recorded 48 h later (6 days after primary infestation) and mean fecundity per surviving female calculated as described above. We also assessed whether immune interference of the field and outbred populations persists following secondary infestation by measuring gene expression on day 6 using the same assay as described above. There were six replicates (*n* = 6) for each pre-infestation treatment divided across seven experimental blocks. Plants pre-infested with the *T. urticae* inducer and *T. evansi* suppressor strains were used as benchmarks for induction and suppression. Uninfested plants were used as a control for the basal level of plant defences. Note that although this was set up in parallel to the experiment measuring gene expression 4 dpi (days post infestation) described above, two additional replicates were performed.

### 2.6. The inbred lines

#### 2.6.1. Genetic variation in *Tetranychus evansi* inbred lines for fecundity on WT and *def-1* tomato plants

We assessed variation in fecundity between lines on the different plant types: in the presence (WT) and absence (*def-1*) of functioning JA immune defences following a similar experimental set-up described for the populations, with some modifications. These were: (i) 12 mated females (13 ± 1 days old) were placed on each leaflet, (ii) leaves were detached from plants with the petiole in water, and (iii) infestations were only for 2 days. Mean fecundity per surviving female was calculated as described above.

Owing to a large number of inbred lines, we randomly tested subsets of inbred lines (incomplete block design). In total, we assessed mite performance across thirty-five temporal blocks over a year. Each block included twelve inbred lines placed on both WT and immunocompromised plants (WT: *n* = 4–7; *def-1*: *n* = 4–7). In each block, there were also two to three replicates of each benchmark control population, i.e. *T. urticae* inducer (WT: *n* = 75; *def-1*: *n* = 72) and *T. evansi* suppressor (WT: *n* = 76; *def-1*: *n* = 67) populations also on both plant types, in addition to clean plants (WT: *n* = 27; *def-1*: *n* = 25).

#### 2.6.2. Expression of tomato defences in response to infestation by *Tetranychus evansi* inbred lines

The expression of marker genes implicated in JA defences (*WIPI-IIc*, *WIPI-IIf*) was measured to assess variation in the defensive response of WT plants for 19 of the inbred lines (*n* = 5–7). The 19 inbred lines that were chosen present the range of fecundity levels observed across the WT and *def-1* plants (electronic supplementary material, figure S1). Induction of defences was also measured for a subset of the benchmark control populations (i.e. inducer and suppressor populations, *n* = 4) and uninfested plants (*n* = 4).

### 2.7. Statistical analysis

All statistical analyses were performed with the software R (version 4.2.2) [54]. All models for gene expression were repeated including or excluding the benchmark controls for immune induction (*T. urticae* inducer), immune suppression (*T. evansi* suppressor) and uninfested plants. This was mostly to ensure that induction of immune defences occurred as in previous experiments [13,46], but also enabled us to test for differences among *T. evansi* populations or inbred lines, i.e. in models excluding benchmark controls.

We fitted two independent generalized linear mixed models (GLMMs) (i.e. one for each host plant type) with a normal error structure (lmer, lme4 package [55]) to investigate whether fecundity (4 dpi) varied among the field and outbred populations on WT and *def-1*. The models included *population* (*T. evansi* Outbred, VIT, 6M1, QG and PBS) as a fixed explanatory variable.

To analyse the transcript accumulation of *effector 84*, we fitted a GLMM with a gamma distribution and a log link function (glmmTMB package [56]). The model included *population* (*T. urticae* inducer, *T. evansi* suppressor, Outbred, VIT, 6M1, QG and PBS) as a fixed explanatory variable. To investigate whether transcript accumulation changed in tomato plants infested with field or outbred populations (4 dpi) and if the levels changed when subsequently infested with the *T. urticae* inducer population (2 days after re-infestation), GLMMs with a gamma distribution and a log link function (glmmTMB package [56]) were fitted for each gene separately (*WIPI-IIc*, *WIPI-IIf*). These models included *population*, *time of gene expression* (4 dpi or 2 days after re-infestation) and their interaction as fixed explanatory variables. glmmTMB models were used instead of glmer to improve model convergence.

To evaluate if the fecundity of *T. urticae* inducer changed following previous infestation with the *T. evansi* field and outbred populations, we fitted a GLMM assuming a Gamma distribution and a log-link function (lme4 package [55]) since normality was not met (Shapiro–Wilk test: *p* = 0.008), and variances were not homogeneous. In these models, the pre-infestation *population* (*T. evansi* Outbred, VIT, 6M1, QG, PBS, and the inducer and manipulator benchmark populations) was included as a fixed explanatory variable.

A GLMM assuming a Gamma distribution and a log-link function (lme4 package [55]) was used to investigate whether there was variation in fecundity of the inbred lines on the different host plant types since normality was not met, but here variances were homogeneous. *Inbred line*, *host plant* (i.e. WT or *def-1*) and their interaction were included in the model as fixed explanatory variables. A similar model was repeated for the 19 inbred lines selected to investigate variation in the induction of plant defences.

We calculated broad-sense heritability (*H*^2^) [57] for the fecundity of the inbred lines on WT and *def-1* by performing separate generalized mixed linear models for each host type with a Gamma distribution and a log link function (lme4 package [55]) with *inbred line* included in the model as a random explanatory variable. From the summary of the models, we extracted the variance for *inbred line*, *block* and *residual variance* of the model and calculated *H*^2^ as follows: $var_{\left(\mathrm{inbred}\;\mathrm{line}\right)}/(var_{\left(\mathrm{inbred}\;\mathrm{line}\right)}+var_{\left(\mathrm{block}\right)}+var_{\left(\mathrm{residuals}\right)})$. To determine the significance of *H*^2^, we compared models, using ANOVA, including and excluding the *inbred line* random factor.

To analyse if there is variation in the induction of plant defences after infestation with the inbred lines, GLMMs with a gamma distribution and a log link function (glmmTMB package [56]) were fitted for each gene separately (*WIPI-IIc or WIPI-IIf*). *Inbred line* was included in the model as a fixed explanatory variable. glmmTMB models were used instead of glmer to improve the convergence of the models.

To test how JA defences influence *T. evansi* fecundity, a genetic correlation between fecundity on WT and *def-1* plants was performed using a generalized mixed linear model assuming a Gamma distribution and a log link function (lme4 package [55]) with *host plant* as a fixed variable and *inbred line* nested within plant (0 + *host plant* | *inbred line*). From the summary of the model, we extracted the correlation obtained in the random effects section. To determine the significance of the genetic correlation, we compared models with and without the correlation using an ANOVA.

We investigated whether the fecundity of the inbred lines on WT plants and on *def-1* plants correlated with normalized gene expression for the defence genes *WIPI-IIc* and *WIPI-IIf* using Pearson correlations [58]. Normalized gene expression was log-transformed to improve the normality of data.

For all analyses, block was included in models as a random variable. For each model, when significant differences were found, multiple comparisons were performed using estimated marginal means (emmeans, emmeans package [59]) and the *p*-values corrected using the false discovery rate method (*α* = 0.05) [60].

## 3. Results

### 3.1. Fecundity, expression of salivary *effector 84* and plant defences after infestation with *Tetranychus evansi* field and outbred populations

**Figure 1.**
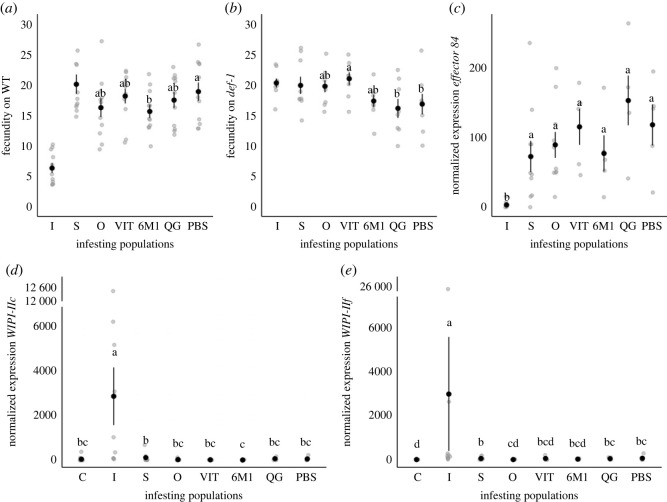
Phenotypic characterization of the field populations and the outbred population. Mean fecundity (± s.e.) of each population on (*a*) WT and (*b*) *def-1* plants at 4 dpi, and the mean normalized gene expression (± s.e.) of (*c*) salivary *effector 84,* and JA-related genes (*d*) *WIPI-IIc* and (*e*) *WIPI-IIf* at 4 dpi. The black circle denotes the mean and each grey circle a replicate. Different lowercase letters indicate statistical differences among populations according to multicomparison analysis performed using estimated marginal means. The benchmark control treatments are denoted as ‘I' for the *T. urticae* inducer control, ‘S' for the *T. evansi* suppressor control, and ‘C' for uninfested plants.

There was variation in levels of fecundity among field and outbred populations on WT (*population*: $\chi_{4}^{2}=12.204$, *p* = 0.016; figure 1*a*) and on *def-1* (*population*: $\chi_{4}^{2}=15.928$, *p* = 0.003; figure 1*b*) plants. On WT, PBS had the highest fecundity, while 6M1 had the lowest. On *def-1*, VIT had the highest fecundity and differed significantly from PBS and QG. Fecundity of the outbred population did not differ from any of the field populations on either plant type.

There was a significant effect of population on the expression of salivary effector 84 ($\chi_{6}^{2}=116.290$, *p* < 0.001; figure 1*c*). This was mainly explained by low levels of expression of this effector for the *T. urticae* inducer benchmark control, with all *T. evansi* populations having similar levels of expression. There was no difference in transcript accumulation levels among *T. evansi* populations.

We observed differences between populations for *WIPI-IIc* ($\chi_{7}^{2}=64.097$, *p* < 0.001; figure 1*d*) and *WIPI-IIf* ($\chi_{7}^{2}=53.344$, *p* < 0.001; figure 1*e*), in analyses including the benchmarks, with both JA marker genes being induced by *T. urticae* inducer. Expression levels of JA marker genes in plants infested with field or outbred populations were similar to defence levels found in plants infested with the *T. evansi* suppressor control, and sometimes to levels observed for uninfested plants (i.e. all populations for *WIPI-IIc* and VIT and 6M1 for *WIPI-IIf*).

### 3.2. Impact of infestation with the field and outbred populations on a JA-susceptible *Tetranychus urticae* population

**Figure 2.**
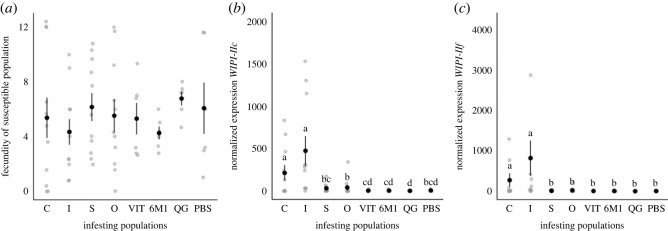
Effect of the prior infestation with *T. evansi* populations on a JA-susceptible and inducer *T. urticae* population. Mean (*a*) fecundity (± s.e.) 2 days after re-infestation and mean normalized gene expression (± s.e.) of JA-related genes (*b*) *WIPI-IIc* and (*c*) *WIPI-IIf* 2 days after re-infestation. Each grey circle represents a replicate and the black circle the mean. Different lowercase letters indicate statistical differences between populations according to multicomparison analysis performed using estimated marginal means. The benchmark control treatments are denoted as ‘I' for the *T. urticae* inducer mite population, ‘S' for the *T. evansi* suppressor population control, and ‘C' for uninfested plants.

Fecundity (2 days after re-infestation) of the JA-susceptible *T. urticae* inducer population was not affected by pre-infestation with any of the field, outbred populations, or benchmark controls (*population*: $\chi_{7}^{2}=7.7907$, *p* = 0.3514; figure 2*a*). However, the expression of JA marker genes changed following secondary infestation depending on the pre-infestation treatment (*population* × *time of gene expression analysis*; *WIPI-IIc*: $\chi_{7}^{2}=24.516$, *p* = 0.001; *WIPI-IIf:* $\chi_{7}^{2}=56.020$, *p* < 0.001; figure 2*b*,*c*). The expression of these genes only increased in previously uninfested plants and plants pre-infested with the inducer benchmark control population. By contrast, plants pre-infested with each of the *T. evansi* populations had no increase in expression, with levels remaining the same or being lower than at 4 dpi.

### 3.3. Genetic variation in *Tetranychus evansi* inbred lines for fecundity and expression of plant defences

**Figure 3.**
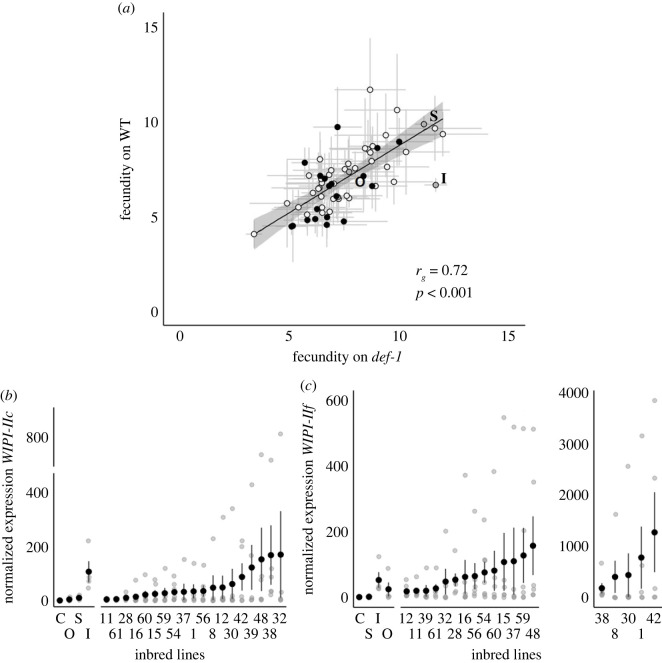
Phenotypic variation of the 59 inbred lines. (*a*) Genetic correlation between mean fecundity (± s.e.) on WT and *def-1.* Black points correspond to the 19 inbred lines selected to study the induction of defences. The black line represents the correlation between traits, excluding benchmark controls, and the grey shadow the 95% confidence intervals. Mean normalized gene expression (± s.e.) of JA-related genes (*b*) *WIPI-IIc* and (*c*) *WIPI-IIf* on WT plants. The 19 inbred lines selected to measure the response of the JA marker genes after infestation are highlighted in black in (*a*). Panel (*c*) has two graphs, to account for the high variation in normalized gene expression of *WIPI-IIf* among lines. Each grey circle represents one replicate. The benchmark control treatments are denoted as ‘I' for the benchmark inducer population, ‘S' for the benchmark suppressor population, ‘O' for the outbred population, and ‘C' for uninfested plants.

There were significant differences among inbred lines for fecundity (*inbred line*: $\chi_{58}^{2}=156.611$, *p* < 0.001; electronic supplementary material, figure S1*a*) with overall higher fecundity on the immunocompromised plants than on WT (*host plants*: $\chi_{1}^{2}=5.371$, *p* = 0.021; electronic supplementary material, figure S1*a*). The interaction between inbred line and host plant was not significant (*inbred line* × *host plants*: $\chi_{58}^{2}=42.549$, *p* = 0.936). The genetic component for this observed phenotypic variation in fecundity was similar on both WT (*H*^2^ = 0.094, $\chi_{1}^{2}=75.435$, *p* < 0.001) and *def-1* (*H*^2^ = 0.1217, $\chi_{1}^{2}=136.490$, *p* < 0.001) plants. There was a strong positive genetic correlation for fecundity in the presence and absence of JA defences across inbred lines (*r*_*g*_ = 0.72, *p* < 0.001; figure 3*a*).

On the subset of 19 inbred lines selected to study the expression of JA plant defences (highlighted in black in electronic supplementary material, figure S1*a*), fecundity varied among inbred lines but did not differ on WT or *def-1* plants (*inbred line*: $\chi_{18}^{2}=45.952$, *p* < 0.001; *host plants*: $\chi_{1}^{2}=3.232$, *p* = 0.072; *inbred line* × *host plants*: $\chi_{18}^{2}=18.235$, *p* = 0.440).

There were differences in levels of expression for *WIPI-IIc* ($\chi_{22}^{2}=78.470$, *p* < 0.001; figure 3*b*) and *WIPI-IIf* (*WIPI-IIf*: $\chi_{22}^{2}=98.584$, *p* < 0.001; figure 3*c*), with the *T. urticae* inducer inducing the highest and the *T. evansi* suppressor showing lower levels of induced defences. *Post* *hoc* comparisons (electronic supplementary material, table S2) revealed that most of the *T. evansi* inbred lines (17/19) had similar levels of *WIPI-IIc* expression to the suppressor control, with 7/19 lines also not differing from uninfested plants. For *WIPI-IIf,* levels of induction for most inbred lines did not differ from either of the benchmark controls (15/19), with 18/19 *T. evansi* inbred lines not differing from the *T. urticae* inducer benchmark. However, only three inbred lines (lines 1, 8 and 42) had higher levels of expression than the *T. evansi* suppressor benchmark. In analyses excluding the benchmark controls, there were also differences among lines for the expression of each gene (*WIPI-IIc:* $\chi_{18}^{2}=32.119$, *p* = 0.021; *WIPI-IIf:* $\chi_{18}^{2}=54.515$, *p* < 0.001).

### 3.4. Influence of JA defences on *Tetranychus evansi* fecundity

There was no phenotypic correlation between the fecundity of the 19 inbred lines on WT plants and the normalized gene expression for either of the genes implicated in JA defences after mite infestation (*WIPI-IIc*: *r* = −0.079, *t*_107_ = −0.821, *p* = 0.414; *WIPI-IIf*: *r* = 0.077, *t*_107_ = 0.811, *p* = 0.419; electronic supplementary material, figure S2*a*,*b*). The same was observed for the correlation between the fecundity of the 19 inbred lines on *def-1* and the normalized gene expression for either of the genes (*WIPI-IIc*: *r* = −0.052, *t*_17_ = −0.214, *p* = 0.833; *WIPI-IIf*: *r* = −0.093, *t*_17_ = −0.386, *p* = 0.704; electronic supplementary material, figure S3*a*,*b*).

## 4. Discussion

We investigated intraspecific variation, in different field populations and inbred lines derived from these populations, for the ability to interfere with host immune responses and how this relates to fecundity. We found no differences among four field populations in the gene expression of *effector 84* and the immune interference of JA defences, despite differences in fecundity among populations on WT and *def-1* plants. We did though find genetic variation among the inbred lines for oviposition on WT plants and variation in induction of gene expression for both marker genes implicated in the JA pathway (*WIPI-IIc* and *WIPI-IIf*). We also found that overall, the different lines had higher fecundity on immunocompromised host plants, indicating that the active JA pathway in WT plants negatively affects *T. evansi* mites. However, higher levels of fecundity in the inbred lines did not correlate with lower levels of immune defences (i.e. higher levels of immune interference).

### 4.1. The fecundity of *Tetranychus evansi* does not correlate with levels of immune interference

JA defences reduced the fecundity of the inbred lines of *T. evansi* as, overall, fecundity was higher on *def-1* than on WT plants. Indeed, plants with a functioning immune response are a more hostile environment for several herbivores, as has been shown for mites and caterpillars [13,48,49,61]. Similarly, plants pre-infested by *T. evansi* have generally been found to increase the fecundity of both *T. evansi* and *T. urticae* [12,13,36,39] although this effect can depend on the timing of the co-infestation [62]. Taken together with our results, it seems that immunocompromised plants, either artificially or via herbivore immune interference mechanisms, confer obvious benefits to arthropod herbivores feeding on them, including *T. evansi* (as shown here with lines laying, on average, 1.07-fold more eggs per female on immunocompromised plants). However, despite genetic variation for the propensity of the *T. evansi* inbred lines to induce immune responses, we did not find a correlation between the degree of immune interference and fecundity.

Fecundity is often used as a proxy for performance, is heritable, and can be influenced by the environment [63–65]. In a sister species, *T. urticae,* fecundity has been observed to have variable levels of narrow-sense heritability (*h*^2^ = 0.72 [63]; *h*^2^ = 0.11 [64]; *h*^2^ = 0.05 [65]). Although these studies revealed genetic and environmental correlations among fecundity and other life-history traits (e.g. development time and juvenile mortality), fecundity was found to be independent of longevity [66] and web production [63]. Similarly, our results suggest that fecundity is independent of the magnitude of JA defences, as heritability was similar on WT and immunocompromised plants and fecundity on WT and *def-1* across lines was positively correlated. It is possible that absolute expression of two marker genes at an arbitrary time point does not adequately reflect the complexity and dynamics of the relevant downstream induced-defence response of tomato plants. Another recent study found that *T. urticae* mites that suppress defensive bubble formation in honeysuckle did not have higher fecundity than inducer mites [67]. In addition, defence induction may affect other traits that were not measured here. It may be that variation in the ability of *T. evansi* to interfere with immune defences is correlated with other life-history traits (longevity, lifetime reproductive performance, juvenile survival, etc.). As such, how other life-history traits covary with immune induction, in longer-term experiments, should be considered in future.

It is generally found that *T. evansi* can suppress tomato immune responses, with corresponding beneficial effects on fecundity, juvenile survival and development rate when compared to *T. urticae* [12,13,36,44,68]. However, previous studies that identified a link between defence induction and herbivore performance included comparisons among either *T. urticae* lines [35] or *Tetranychus* species [13].

It is possible that some of the inbred lines with higher fecundity may be more resistant to tomato defences than other lines. Plant defence resistance has been demonstrated in *T. urticae* whereby some lines induce host immune genes but maintain high levels of fecundity [35,69]. If this is the case, resistance to plant defences may be via detoxification mechanisms (e.g. metabolite modification, degradations and/or secretion) [66,69–71]. One study found *T. urticae* to have increased expression of genes involved in detoxification following only five generations on tomato (a novel stressful environment after bean host plants), with correlated beneficial effects in other toxic environments such as the presence of pesticides [69].

### 4.2. Genetic variation for immune interference among lines, but not among populations

There were differences in immune interference among the different inbred lines, but the four field populations did not differ in the extent to which they upregulated tomato defences. It is most probable that this is because the different populations contain similar levels of genetic variation that was captured across the different lines. This is supported by the observation that the variation in defence gene induction by the outbred population overlaps with variation across inbred lines. Thus, in our lines, we probably fixed the genetic variation present within the field populations.

### 4.3. Immune interference in *Tetranychus evansi* populations and consequences for heterospecifics

Not only the magnitude of defence activation but also the expression levels of *effector 84* were similar across the *T. evansi* field populations. This was despite variation in levels of fecundity among the field populations on both WT and *def-1* plants. *effector 84* is a salivary effector protein that is involved in the suppression of the JA and SA pathways in plants [14,37]. To date, no study has addressed variability in the expression of this effector within the *Tetranychus* genus, as most studies addressing effector genes mainly focus on the mechanism and mode of action of such molecules (reviewed in [21]). The absence of significant variation in the expression of this effector may be explained by the fact that all these populations belong to the same haplotype (ITS lineage I [44]). It is possible that another haplotype (ITS lineage II) may suppress plant defences more strongly [46]. However, Knegt *et al*. [46] also observed little variability in traits related to immune interference across populations. Nevertheless, it would be interesting to assay these populations for variation in the expression of *effector 84* and see how it relates to the expression of plant defence genes and *T. evansi* life-history traits. More studies investigating the dynamics in effector gene expression and linking this to the expression of immune genes in host plants could improve our understanding of the mechanisms of coevolution between plants and their parasites.

We did find that infestation with the *T. evansi* field and outbred populations prevented plants from mounting an effective immune response against the JA defence susceptible *T. urticae* [13]. In plants pre-infested with mites from these *T. evansi* populations, levels of induction for all defence genes were maintained at the same or lower levels after subsequent infestation with the *T. urticae* inducer population. This lasting effect of defence interference could result from a latency period required for the host plant to re-establish its normal defensive status in response to a secondary infection. Moreover, in several co-infection studies when infections are sequential, host-mediated facilitation by a suppressor parasite has been shown to increase fitness-related traits in a second parasite [6,12,13,36,39]. This facilitation may in turn promote competition with herbivores co-habiting the same host (e.g. spider mites [12,43,72]; beetles [73]). Investigating how the lasting effect of defence interference persists through time and its benefits for con- and heterospecific individuals would increase our understanding of how species may evolve in communities. In the experiments presented here, however, despite defences being maintained at low levels, we did not observe facilitation of the JA-susceptible *T. urticae* population in terms of fecundity. Previous studies showed that the oviposition of this heterospecific competitor of *T. evansi* is higher on suppressed and uninfested plants, compared to plants with induced defences [12,13,35,39]. As previously reported, the outcome of facilitation experiments may be variable and strongly depend on the timing of the infestation and the number of mites used [24,36,37,74].

### 4.4. Plasticity of immune interference

We found no evidence for metabolic costs of immune interference as lines showing higher levels of immune interference did not have lower levels of fecundity on *def-1* plants (electronic supplementary material, figure S3). This type of trade-off has been investigated in other parasites, with varying results. Immune interference by a parasitoid was lost following selection on a diversity of hosts, possibly due to being ineffective, and/or costly to maintain on some host species [27]. Another study found metabolic detoxification to trade-off with increased population growth rate in the *Sitobion avanae* aphid [75]. By contrast, no trade-off was found between the growth rate of *Depressaria pastinacella* or *Heliothis zea* caterpillars and the production of detoxification enzymes [76,77]. Also, there was no apparent cost for *Schistocephalus solidus* manipulating the behaviour of its intermediate host (to increase predation by its definitive host [78]). Another study, with *T. evansi,* showed the maintenance of suppression after evolution on immunosuppressed (*def-1*) plants for 60 generations suggesting marginal costs associated with this trait in this species [79]. Low costs for the maintenance of immune interference may be explained through trait plasticity. Indeed, responses to induced plant defences are phenotypically plastic for some herbivores, only being expressed on plants when needed [49,70,80]. For *T. evansi* this seems to be the case, as the *effector 84* was found to be highly plastic in certain conditions (e.g. the presence of heterospecific competitors [37]; light–dark cycles [42]; developmental stages [81]).

Spider mites also show plasticity in other traits related to immune interference [37]. An ecological cost for parasites may be the presence of competitors in the within-host environment that benefit from, but do not contribute to, immune interference [24,26,82]. In the presence of competitors, *T. evansi* mites can increase web density [43], local levels of immune interference and their oviposition [37], showing plasticity in traits facilitating their monopolization of the immunosuppressed environment. This indicates that trait plasticity may help mitigate ecological costs associated with immune interference.

### 4.5. Immune interference as a public good

Immune interference could be seen as a public good, if it benefits other parasites sharing the same host [6,12,13,36,39]. This could lead to the emergence of cheaters/exploiters of the same or different species, which do not suppress defences but have higher fitness when it occurs, since they pay no energetic costs associated with immune interference. A theoretical study revealed that it is possible for two strains with extreme immune interference strategies (i.e. zero and maximum) to coexist in a population [26]. This could produce antagonistic coevolutionary dynamics between parasite strains that can interfere with their host immune system, that strive to monopolize the suppressed environment, and cheaters that aim to reap the benefits [83]. Cheaters that benefit from, but do not contribute to, immune interference have been identified in *Pseudomonas aeruginosa* and *Yersinia pestis* [84]. The emergence of cheaters exploiting public goods has also been identified *in vitro* in the bacterium *Pseudomonas flourescens* [85,86]. Selection for suppression and cheating would be possible in *T. evansi* considering the potential for genetic variation of this trait. However, support for this hypothesis in this system would require identifying the (benefits) and costs of immune interference either energetically (i.e. via the production of interfering molecules, such as salivary effectors), or the presence of competitors exploiting and benefitting from the resource more than suppressor lines. It would be interesting to establish the relationship or co-occurrence between the fecundity of putative cheaters and their proximity to suppressors in natural populations.

The fact that suppression enables overcoming host immune responses and can be beneficial for competitor parasites of the same or different species, means this trait may coevolve in response to both the host and other parasites [7,25]. These may be important factors responsible for the maintenance of genetic variation in this trait. For instance, selection for immune interference may depend on the host environment, and the frequency at which parasites encounter hosts upon which immune interference is effective, or in co-infections with other parasites that exploit the manipulated host environment.

## 5. Conclusion and perspectives

Our results show genetic variation for fecundity within a *T. evansi* population, but this does not correlate with variation in levels of induction of immune defences. This might be because intraspecific variation for immune interference might be linked to other *T. evansi* life-history traits that, for example, are more targeted by host immune defences. We advocate that more studies should be conducted to investigate the presence and causes of intraspecific variation for immune interference and consequences for parasite life-history traits, in both the absence and presence of competitors. This should contribute to a better understanding of how and when variation in traits related to immune interference may be maintained in parasite populations, its role as a driver for coevolution with hosts and competitors and how it relates to outbreaks of parasites or pest species.

## Data Availability

Data are available in an open-access data repository: https://doi.org/10.5061/dryad.hmgqnk9mx [87]. The data are provided in electronic supplementary material [88].
